# Attitudes toward psychedelics and psychedelic-assisted therapy among potential mental health service users and the general population in Australia

**DOI:** 10.1177/00048674241261779

**Published:** 2024-06-22

**Authors:** Zohaib Nadeem, Stephen Parker, Hugh McGovern, Lena KL Oestreich

**Affiliations:** 1 Faculty of Medicine, The University of Queensland, Brisbane, QLD, Australia; 2 Metro North Mental Health, Royal Brisbane and Women’s Hospital, Herston, QLD, Australia; 3 Faculty of Medicine, Griffith University, Brisbane, QLD, Australia; 4 The Cairnmillar Institute, Melbourne, VIC, Australia; 5 School of Psychology, The University of Queensland, Brisbane, QLD, Australia; 6 Centre for Advanced Imaging (CAI) and Australian Institute for Bioengineering and Nanotechnology (AIBN), The University of Queensland, Brisbane, QLD, Australia; 7 National Imaging Facility, The University of Queensland, Brisbane, QLD, Australia

**Keywords:** Psilocybin, psychedelics, psychedelic-assisted therapy, treatment-resistant depression, MDMA

## Abstract

**Objective:**

Despite rapid advances in psychedelic sciences and the increasing number of countries legalizing psychedelics for the treatment of mental illnesses, the attitudes, knowledge and readiness of both mental health consumers and the general population remain largely unknown.

**Methods:**

A cross-sectional survey was conducted among Australians, targeting individuals with mental illness as potential mental health service users. A sub-sample of individuals free of mental illness was also surveyed to assess attitudes in the general population. Participants completed the Attitudes on Psychedelics Questionnaire, the Basic Knowledge of Psychedelics Test and a questionnaire by Corrigan et al. to capture attitudes toward psychedelic therapy by mental health service users.

**Results:**

Of the 502 respondents, 64.5% self-identified as having a mental illness. A significant proportion favored legalizing psychedelics for medical use (43%) and were open to their use (52.4%), yet fewer viewed their effects positively (24%) or considered them safe (33%). Most participants reported to be psychedelic naive (61%). Participants with mental illness had significantly more experience with psychedelics than participant free of mental illness (44.1% vs 29.7%). Experience, perceived knowledge and actual knowledge significantly predicted attitudes toward legalization, effects, risks and openness to psychedelics.

**Conclusions:**

While a large proportion of Australians are in favor of legalizing psychedelics for medical purposes, concerns about safety remain. People with self-identified mental illness, those with previous recreational psychedelic experience and those with greater knowledge of psychedelics were more likely to have positive attitudes toward psychedelics and psychedelic-assisted therapy.

## Introduction

Psychedelics are substances known for their hallucinogenic and consciousness-altering effects, which have been primarily attributed to their action as agonists at 5-hydroxytryptamine 2A (5-HT_2A_) serotonin receptors (Nichols, 2016). Classic examples of psychedelics include lysergic acid diethylamide (LSD), psilocybin, dimethyltryptamine (DMT), mescaline, peyote and ayahuasca (Nichols, 2016). While entactogens, such as 3,4-methylenedioxymethamphetamine (MDMA), can exhibit similar hallucinogenic properties, they are pharmacologically distinct, acting on serotonin, dopamine and norepinephrine pathways (Nichols, 2016; Sarparast et al., 2022). The shared consciousness-altering characteristics of classic psychedelics and MDMA are the focus of their therapeutic applications.

While evidence suggests that humans have used psychedelics for more than 5,000 years, Western science only began to consider these substances in the late 19th century (Rucker et al., 2018; Samorini, 2019). This initial interest was followed by an explosion of research into their psychiatric applications, particularly during the 1950s and 1960s (Rucker et al., 2018). However, a cultural shift in attitudes and government policies in the early 1970s led to the stagnation of psychedelic research (Nichols, 2016; Rucker et al., 2018). In recent years, there has been a resurgence of interest in the potential of psychedelics as treatments for mental illnesses (Nichols, 2016; Rucker et al., 2018). Recent randomized controlled trials of psilocybin have demonstrated rapid and sustained relief from symptoms in individuals with treatment-resistant major depressive disorder (MDD) (Andersen et al., 2021; Goodwin, 2023; Haikazian et al., 2023; Muttoni et al., 2019; Raison et al., 2023). Furthermore, a phase-3 clinical trial has suggested that MDMA may be effective in treating post-traumatic stress disorder (PTSD) (Mitchell et al., 2023).

This emerging evidence led the Therapeutic Goods Administration (TGA) in Australia to approve the use of MDMA for the treatment of PTSD and psilocybin for interventions in treatment-resistant depression (The Therapeutic Goods Administration, 2023). However, this decision has been met by skepticism by many who argue that the policy was implemented hastily without adequate input from experts in the field (Hall and Humphreys, 2022; Kisely, 2023). Concerns have been raised about the lack of comprehensive evidence for the safety and effectiveness of these treatments (McNamee et al., 2023; Reiff et al., 2021), the absence of clear protocols for practitioners (Aday et al., 2022; Schatzberg, 2020) and the need for oversight to support equitable access (Haridy, 2023; Kisely, 2023; Nogrady, 2023).

Classified as illegal narcotics in most countries, psychedelics are often associated in public perceptions with criminal behavior, counter-cultural movements and psychotic states (Rucker et al., 2018). The recreational use of psychedelics is not without its hazards, including issues related to dosage, interactions with other drugs, impaired decision-making, alterations in personality and the potential for psychosis in individuals with predispositions (Anderson et al., 2020; Nichols, 2016). Researchers caution against overly optimistic views on the therapeutic benefits of psychedelics (Anderson et al., 2020; Yaden et al., 2021). Clinical research on psychedelics also faces significant challenges, including ethical dilemmas around the ability of psychiatric patients to give informed consent, difficulties in measuring treatment outcomes and technical hurdles in designing randomized controlled trials (Rucker et al., 2018).

It is likely that certain stigmas and misconceptions about psychedelics persist (Belouin and Henningfield, 2018; Kunstler et al., 2023). The impact of these stigmas on an individual’s willingness to pursue psychedelic therapy remains unclear. Previous surveys on attitudes toward psychedelics have primarily focused on specific groups, such as college students, psychiatrists and psychologists (Kucsera et al., 2023; Wildberger et al., 2017). Results generally indicate cautious yet substantial support for researching the potential of psychedelics in treating psychiatric conditions. Nonetheless, the broader public’s perception of psychedelics, particularly in Australia, is still largely unexplored.

As interest and actions toward expanding psychedelic treatment availability in Australia progresses, there is an urgent need to understand the values and needs of all stakeholders, including healthcare recipients (Adhikari et al., 2020). This is particularly crucial in the context of psychedelic research, to prevent a repeat of the historical stigmatization of psychedelics and regulatory bans (Yaden et al., 2021). This study aims to survey attitudes toward psychedelics among potential mental health service users and individuals without any diagnosed mental illness in Australia.

## Methods

A cross-sectional survey method was employed. The University of Queensland Human Research Ethics Committee A granted ethical approval for the anonymous online survey (Application ID: 2007718). The methodology and findings are reported in accordance with the Checklist for Reporting of Survey Studies (CROSS, see Supplementary Material 1).

### Participants

Participants were recruited via the Prolific research platform from 13 September 2023 to 14 November 2023 and were compensated at a rate of AU$20 per hour. Two groups were targeted: (1) individuals who self-reported a history of mental illness (MI+) and (2) individuals who reported no current or past mental illness (MI−). We aimed to have a minimum sample size of 139 participants per group. This sample size was calculated to ensure sufficient power using analysis of covariance (ANCOVA) to detect small to medium effect sizes (Cohen’s *d* = 0.2–0.5) with a power of 0.95 at an alpha level of 0.05. Eligibility criteria for participation in this study included being 18 years or older, able to provide informed consent, proficient in English, and a resident of Australia.

### Design and questionnaires

Participants completed online questionnaires on their computers or mobile devices. The Attitudes on Psychedelics Questionnaire (APQ) was used to evaluate participants’ views on the legal use of psychedelics, their effects and associated risks (Žuljević et al., 2022). The APQ has previously demonstrated excellent reliability and convergent validity (Žuljević et al., 2022). Participants rated the questions on a 5-point Likert-type scale ranging from 1 (completely disagree) to 5 (completely agree). In addition, the Basic Knowledge of Psychedelics Test (BKPT) (Žuljević et al., 2022) was used to assess participants’ ability to accurately classify substances as psychedelics. Furthermore, a questionnaire developed by Corrigan et al. (2022) was utilized to explore aspects related to psychedelic substances, psychedelic therapies and general substance use. This instrument consists of questions rated on a 5-point Likert-type scale, ranging from 1 (strongly disagree) to 5 (strongly agree).

### Data availability statement

The data used in this study are available from the corresponding author upon request.

### Data analysis

All statistical analyses were completed using SPSS Statistics, version 29.0.1.0 and RStudio 2023.12.0 (IBM Corp, 2023; RStudio Team, 2023). Composite scores were assessed using parametric tests, with non-parametric tests applied where the Kolmogorov–Smirnov test indicated deviations from normality. Demographic variables were compared between groups using chi-square tests. Where significant differences were found, these variables were included as covariates in subsequent analyses.

The ‘actual knowledge of psychedelics’ score was quantified from the BKPT as the number of correctly identified psychedelics minus the number of incorrectly identified psychedelics. The ‘perceived knowledge of psychedelics’ score was derived from three items on the Corrigan scale assessing self-rated knowledge about psilocybin and MDMA. Scores were normalized to a 0–100 scale using:

$$x=\frac{S-m}{M-m}\times 100$$

where *S* is the total score, *m* is the minimum theoretical value and *M* is the maximal theoretical value.

Knowledge, experience and attitude differences were explored via ANCOVA with *knowledge* (actual, perceived), *drug experience* (psilocybin, LSD, mescaline or peyote, salvia, microdosing, ketamine, tobacco, alcohol, cannabis, cocaine [powder], crack cocaine, ecstasy, MDMA, amphetamines, methamphetamines, barbiturates, benzodiazepines, Gamma-Hydroxybutyrate (GHB) or Gamma-Butyrolactone (GBL) other hypnotics or sedatives, volatile inhalators, opioids, anabolic steroids) or *attitudes* (legalization of psychedelics, effects of psychedelics, risk of psychedelics, openness to psychedelics) as the within-subjects factor and *group* (MI+, MI−) as between-subjects factor. Partial correlations were conducted between perceived and actual knowledge of psychedelics by group. Regression analyses were conducted to assess whether knowledge and experience of psychedelics could predict attitudes toward the legal use of psychedelics, effects of psychedelics, risk of psychedelics and openness to psychedelics, while controlling for covariates.

Repeated-measures ANCOVAs were performed to assess attitudes specific to psilocybin and MDMA, assessing factors such as *mental health conditions*, *beliefs about psychedelics*, and *safety* and *legality concerns*. All analyses were Bonferroni-corrected, with the number of comparisons used to calculate the corrected *p*-value stated. Partial eta squared (η_p_^2^) was used as effect size measure.

## Results

Of the 521 participants who started the survey, 19 were excluded due to incomplete responses, resulting in a final sample size of 502 participants. On average, survey completion took 13 minutes and 58 seconds (*SD* = 8.06 minutes). The response rate was 31%, based on the number of participants meeting the inclusion criteria on Prolific at the time of testing.

### Demographics

The average age of participant was 33.46 years (*SD* = 10.84, range = 18–86 years), with the majority rating their natal sex as female (59.4%, *n* = 298) and 40.6% (*n* = 204) as male. In addition, 2.8% (*n* = 14) reported their gender identity as non-binary and 0.8% (*n* = 4) as transgender male. Participants affected by mental illness (MI+) comprised 64.5% (*n* = 324) of the sample (see Table 1). Three participants (0.6%) did not respond to the question regarding their history of mental illness and were excluded from group comparisons.

**Table 1.** Demographics.

	Mental illness	No mental illness	Comparison
Age, mean (SD)	32.53 (9.71)	34.77 (11.9)	*t* **(497)** = **2.27**, *p* = **0.024**
Natal sex, *n* (%)			***χ***^2^ **(1)** = **24.99**, *p* **<** **0.001**
Female	219 (67.6)	78 (44.6)	***ASR*** = **-5**, *p* **<** **0.001**
Male	105 (32.4)	97 (55.4)	***ASR*** = **5**, *p* **<** **0.001**
Gender identity,^a^ *n* (%)			*χ*^2^(1) = 1, *p* = 0.316
Non-binary	13 (4)	1 (0.6)	*ASR* = -1, *p* = 0.635
Transgender male	3 (0.9)	1 (0.6)	*ASR* = 1, *p* = 0.635
Education,^b^ *n* (%)			***χ***^2^ **(8)** = **19.7**, *p* = **0.012**
Left school without qualifications (left before year 10)	3 (0.9)	0	*ASR* = -1.3, *p* = 1
Some high school (completed some year 10)	1 (0.3)	0	*ASR* = -0.7, *p* = 1
Completed high school (completed year 10)	20 (6.2)	5 (2.9)	*ASR* = -1.6, *p* = 0.986
Some college (years 11 and 12)	7 (2.2)	4 (2.3)	*ASR* = 0.1, *p* = 1
Completed college (completed year 12)	37 (11.4)	30 (17.1)	*ASR* = 1.8, *p* = 0.646
Some university (completed some bachelor’s degree)	80 (24.7)	25 (14.3)	*ASR* = -2.7, *p* = 0.062
Completed university (completed bachelor’s degree)	99 (24.7)	65 (37.1)	*ASR* = 1.5, *p* = 1
Some post-graduate degree (completed some of a master’s/PhD)	33 (10.2)	11 (6.3)	*ASR* = -1.5, *p* = 1
Completed post-graduate (completed master’s/PhD)	44 (13.6)	35 (20)	*ASR* = 1.9, *p* = 0.517
Employment,^c^ *n* (%)			***χ***^2^ **(4)** = **24.16**, *p* **<** **0.001**
Student	46 (14.2)	23 (13.1)	*ASR* = -0.3, *p* = 1
Unemployed	54 (16.7)	10 (5.7)	***ASR*** = **-3.5**, *p* = **0.002**
Part-time job	80 (24.7)	29 (16.6)	*ASR* = -2.1, *p* = 0.177
Full-time job	134 (41.4)	108 (61.7)	***ASR*** = **4.4**, *p* **<** **0.001**
Retired	10 (3.1)	4 (2.3)	*ASR* = -0.5, *p* = 1
Religion,^d^ *n* (%)			*χ*^2^(6) = 14.89, *p* = 1
Christian (all denominations)	57 (17.6)	43 (26.6)	*ASR* = 1.9, *p* = 0.46
Muslim	1 (0.3)	5 (2.9)	*ASR* = 2.5, *p* = 0.1
Buddhist	9 (2.8)	3 (1.7)	*ASR* = -0.7, *p* = 1
Hindu	4 (1.2)	6 (3.4)	*ASR* = 1.7, *p* = 0.713
Jewish	2 (0.6)	2 (1.1)	*ASR* = 0.6, *p* = 1
Sikh	0	0	
I am not religious	236 (72.8)	111 (63.4)	*ASR* = -2.2, *p* = 0.222
Other	15 (4.6)	5 (2.9)	*ASR* = -1.0, *p* = 1

SD: standard deviation; ASR: adjusted standardized residual.

*p*-values of post hoc comparisons are Bonferroni corrected.

a Bonferroni-corrected *p*-value for two comparisons.

b Bonferroni-corrected *p*-value for five comparisons.

c Bonferroni-corrected *p*-value for five comparisons.

d Bonferroni-corrected *p*-value for eight comparisons.

The MI− group was significantly older (*M_difference_* = 2.24, *SE_difference_* = 0.99) and had a lower proportion of female participants (44.6%) compared with the MI+ group (67.7%). Although the chi-square test for educational attainment was significant between the two groups, post hoc tests did not remain significant after Bonferroni correction for multiple comparisons (nine comparisons). Employment status differed significantly between the groups, with the MI+ group having a higher proportion of unemployed participants (16.7%) compared with the MI− group (5.7%). Conversely, a smaller proportion of the MI+ group reported being full-time employed (41.4%) compared with the MI− group (61.7%). Consequently, age, natal sex, educational attainment and employment status were included as covariates in subsequent analyses.

### Mental illness

**Figure 1.**
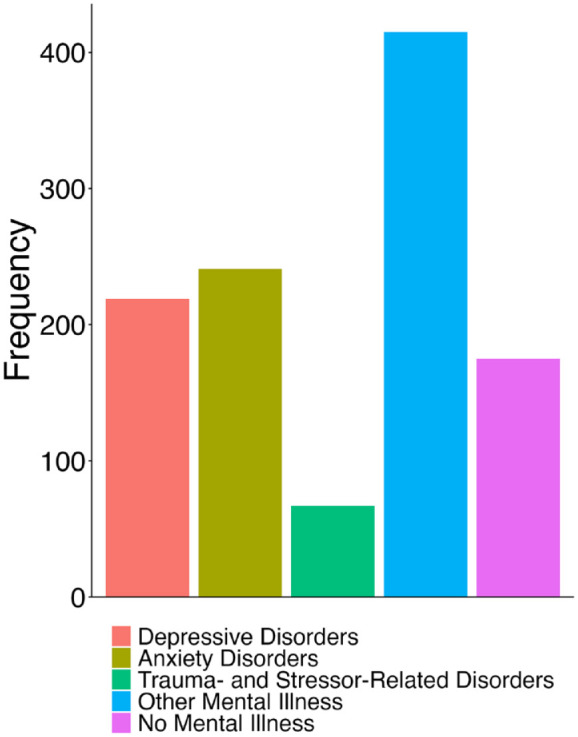
Frequency of mental illnesses throughout the sample. Disorders are classified into broader categories.

The most commonly reported mental illnesses were generalized anxiety disorder (70.7%), major depression (68%) and post-traumatic stress disorder (19.4%; see Figure 1 and Supplementary Figure 1 for a more detailed breakdown of mental illnesses). Comorbidity of mental health diagnoses was common, occurring in 68% of the MI+ group.

### Knowledge of psychedelics

The two groups did not differ significantly in their knowledge of psychedelics, *F*(5,491) = 1.16, *p* = 0.326, η_p_^2^ = 0.012. Across both groups, the three psychedelics most correctly recognized were LSD (MI+: 88.9%, MI−: 78.9%), psilocybin (MI+: 82.4%, MI−: 71.4%) and MDMA (MI+: 68.8%, MI−: 76%). Both groups most commonly misidentified ketamine (MI+: 42.9%, MI−: 32.6%) and opium (MI+: 35.8%, MI−: 42.3%) as psychedelics. Other substances frequently misidentified as psychedelics included GHB (MI+: 27.5%, MI−: 28%) and methamphetamine (MI+: 23.5%, MI−: 33.1%). Responses for each substance by group are detailed in Supplementary Table 1. A significant negative partial correlation was found between perceived and actual knowledge about psychedelics, but only in the MI+ group (*r* = −0.13, *p* = 0.023). This indicates that in individuals with mental illness, actual knowledge of psychedelics decreases as self-rated knowledge about psychedelics increases (see Supplementary Figure 2).

### Experience with psychedelics and other drugs

Most of the sample had never used psychedelics (*n* = 306, 61%); 9.8% (*n* = 49) of the sample indicated that they had used psychedelics once, 16.9% (*n* = 85) reported using psychedelics 2–5 times, 9.2% (*n* = 46) reported using them 6–20 times and 3.2% (*n* = 16) more than 20 times. A repeated-measures ANCOVA found main effects for *drug*, *F*(21,10269) = 16.46, *p* < 0.001, η_p_^2^ = 0.033, and *group*, *F*(1,489) = 10.81, *p* = 0.001, η_p_^2^ = 0.022, as well as a *drug* × *group* interaction, *F*(21,10269) = 3.62, *p* < 0.001, η_p_^2^ = 0.007. Bonferroni-corrected post hoc tests (22 comparisons) found that, relative to the MI− group, the MI+ group had significantly more experience using cannabis, *F*(1,489) = 8.73, *p* = 0.003, η_p_^2^ = 0.018, amphetamines, *F*(1,489) = 13.12, *p* < 0.001, η_p_^2^ = 0.026, methamphetamines, *F*(1,490) = 5.71, *p* = 0.017, η_p_^2^ = 0.012, benzodiazepines, *F*(1,490) = 7.61, *p* = 0.006, η_p_^2^ = 0.015, opioids, *F*(1,489) = 15.25, *p* < 0.001, η_p_^2^ = 0.03, LSD, *F*(1,489) = 5.27, *p* = 0.022, η_p_^2^ = 0.01, and tobacco, *F*(1,489) = 10.14, *p* = 0.002, η_p_^2^ = 0.02.

### Attitudes to psychedelics (APQ)

Overall, a large proportion of participants were ‘undecided’ about the legalization of psychedelics, their effects, safety and their openness to using psychedelics, with percentages of 39%, 38%, 48% and 30%, respectively. While a larger proportion of participants favored the legalization of psychedelics for medical purposes (43% agreed or strongly agreed vs 17% disagreed or strongly disagreed) and indicated their openness to using psychedelics (52.4% agreed or strongly agreed vs 17% disagreed or strongly disagreed), they were less likely to rate the effects of psychedelics positively (24% agreed or strongly agreed vs 38% disagreed or strongly disagreed) or to perceive the use of psychedelics as safe (33% agreed or strongly agreed vs 19% disagreed or strongly disagreed).

**Figure 2.**
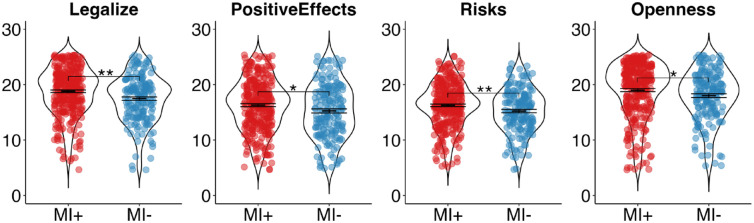
Group comparisons on attitudes toward psychedelics. MI+: Mental Illness group; MI−: No Mental Illness group; *p < 0.05, **p < 0.01, ***p < 0.001. Error bars represent the standard error of the mean.

A main effect for *group*, *F*(1,484) = 8.61, *p* = 0.004, η_p_^2^ = 0.017, was observed. Bonferroni-corrected post hoc tests (four comparisons) revealed that, relative to the MI− group, the MI+ group was significantly more in favor of legalizing psychedelics, *F*(1,484) = 11.16, *p* < 0.001, η_p_^2^ = 0.023, rated the effects of psychedelics as significantly more positive, *F*(1,484) = 4.93, *p* = 0.027, η_p_^2^ = 0.01, perceived psychedelics as having significantly fewer risks, *F*(1,484) = 8.11, *p* = 0.005, η_p_^2^ = 0.016, and were significantly more open to psychedelics, *F*(1,484) = 5.68, *p* = 0.018, η_p_^2^ = 0.012 (see Figure 2).

### Effects of experience and knowledge on attitudes to legal use of psychedelics, effects of psychedelics, risk of psychedelics and openness to psychedelics

Variance inflation factors (VIFs) were below two for both covariates and independent variables in all models, indicating no concerns with multicollinearity. *p*-values from regression analyses were adjusted using Bonferroni correction for four analyses. Multiple linear regression analyses with the independent predictors ‘perceived knowledge of psychedelics’, ‘actual knowledge of psychedelics’ and ‘past experience with psychedelics’ significantly predicted (1) attitudes toward the legalization of psychedelics, (2) perceived effects of psychedelics, (3) perceived risks of psychedelics and (4) openness to psychedelics (see Table 2). The independent predictors consistently contributed significant additional variance, above and beyond the variance explained by the covariates alone. Notably, perceived knowledge about psychedelics emerged as a strong predictor across all models, with standardized coefficients indicating a robust relationship with each outcome measure. These findings suggest that while demographic factors and general education level provide a foundational understanding, specific knowledge and perceptions about psychedelics play a crucial role in shaping individuals’ attitudes toward psychedelics.

**Table 2.** Summary of multiple linear regression analyses.

Predictor	Model 1: attitudes toward legalizing psychedelics β (*p*)	Model 2: perceived effects of psychedelics β (*p*)	Model 3: perceived risks of psychedelics β (*p*)	Model 4: openness to psychedelics β (*p*)
**Step 1**				
Age	-0.044 (0.358)	-0.106 (0.027)*	-0.071 (0.141)	-0.122 (0.011)*
Natal sex	-0.036 (0.439)	-0.036 (0.434)	-0.002 (0.962)	-0.010 (0.825)
Employment	-0.008 (0.874)	0.041 (0.398)	-0.001 (0.983)	0.036 (0.461)
Education	0.093 (0.046)*	0.000 (0.999)	0.017 (0.724)	0.109 (0.019)*
**Step 2**				
Age	-0.006 (0.891)	-0.063 (0.114)	-0.028 (0.501)	-0.088 (0.043)*
Natal sex	-0.022 (0.586)	-0.025 (0.509)	0.009 (0.815)	0.001 (0.982)
Employment	-0.067 (0.126)	-0.040 (0.327)	-0.067 (0.119)	-0.018 (0.694)
Education	0.123 (0.003)**	0.052 (0.186)	0.052 (0.208)	0.136 (0.001)**
Actual knowledge	0.116 (0.004)**	0.121 (0.001)**	0.174 (<0.001)***	0.087 (0.032)*
Experience with psychedelics	0.068 (0.197)	0.200 (<0.001)***	0.120 (0.020)*	0.035 (0.505)
Perceived knowledge	0.421 (<0.001)***	0.425 (<0.001)***	0.418 (<0.001)***	0.411 (<0.001)***
**Model summary**	*R*²_adj_ = 0.216***	*R*²_adj_ = 0.322***	*R*²_adj_ = 0.252***	*R*²_adj_ = 0.196***
	Δ*R*² = 0.215 (<0.001)	Δ*R*² = 0.320 (<0.001)	Δ*R*² = 0.257 (<0.001)	Δ*R*² = 0.184 (<0.001)

β: standardized coefficients; *p: p*-values; *R*²_adj_: adjusted *R*²; Δ*R*²: *R*^2^ change.

Step 1 of each model includes the covariates only. Independent predictors are added in Step 2.

* *p* < 0.05, **p < 0.01, ***p < 0.001.

### Attitudes to psilocybin- and MDMA-assisted psychotherapy

#### Usefulness for treatment

**Figure 3.**
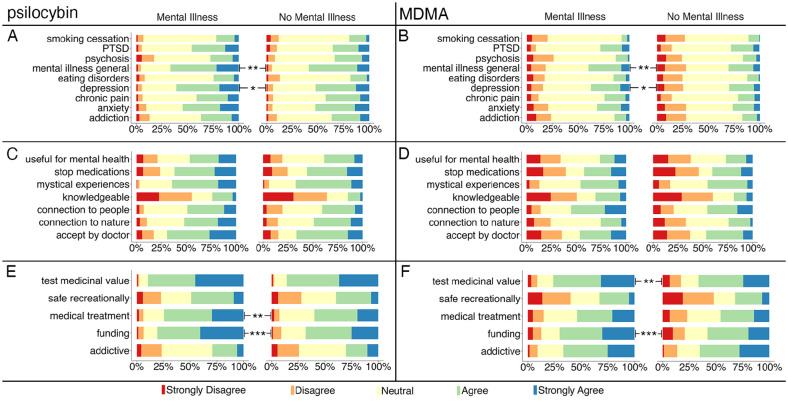
Usefulness for treatments (A, B), beliefs and acceptance (C, D), and safety and legality (E, F) of psilocybin (A, C, E) and MDMA (B, D, F) were compared between Mental Illness and No Mental Illness groups. *p < 0.05, **p < 0.01, ***p < 0.001.

Significant main effects for *condition*, *F*(8,3928) = 3.32, *p* < 0.001, η_p_^2^ = 0.007, and *drug*, *F*(1,491) = 14.74, *p* < 0.001, η_p_^2^ = 0.029, were observed, as well as significant *group* × *condition* interaction, *F*(8,3928) = 4.84, *p* = 0.028, η_p_^2^ = 0.01. Bonferroni-corrected post hoc tests for nine comparisons revealed that the MI+ group was more likely to agree that psilocybin- and MDMA-assisted psychotherapy could be useful for the treatment of mental illnesses in general, *F*(1,491) = 9.67, *p* = 0.002, η_p_^2^ = 0.019, and for depression, *F*(1,491) = 6.7, *p* = 0.01, η_p_^2^ = 0.013, than MI− group (see Figure 3(A) and (B)). Across both groups and conditions, psilocybin was perceived as more useful for treatment than MDMA, *F*(1,491) = 264.36, *p* < 0.001, η_p_^2^ = 0.35.

#### Beliefs and acceptance

Significant main effects for *beliefs*, *F*(6,1968) = 5.69, *p* < 0.001, η_p_^2^ = 0.017, and *drug*, *F*(1,328) = 6, *p* = 0.025, η_p_^2^ = 0.015, were observed. Overall, participants indicated that they were significantly more knowledgeable about MDMA than psilocybin, *F*(1,328) = 5.57, *p* = 0.019, η_p_^2^ = 0.017. Across groups, participants believed that psilocybin increases people’s connection to nature, *F*(1,328) = 77.78, *p* < 0.001, η_p_^2^ = 0.192, and induces mystical experiences, *F*(1,328) = 44.47, *p* < 0.001, η_p_^2^ = 0.119, significantly more than MDMA. Finally, participants from both groups also agreed significantly more that psilocybin, compared with MDMA, would be therapeutically useful for their mental illness, *F*(1,328) = 18.67, *p* < 0.001, η_p_^2^ = 0.054, that they would be more willing to accept psilocybin with psychological support if a doctor recommended it, *F*(1,328) = 38.33, *p* < 0.001, η_p_^2^ = 0.105, and that they would be more willing to gradually cease their medications to receive psilocybin-assisted psychotherapy as opposed to MDMA-assisted psychotherapy, *F*(1,328) = 25.7, *p* < 0.001, η_p_^2^ = 0.073.

#### Safety and legality

Significant main effects for *safety*, *F*(4,1964) = 6.32, *p* < 0.001, η_p_^2^ = 0.013, and *group*, *F*(1,491) = 12.16, *p* < 0.001, η_p_^2^ = 0.024, were observed, along with significant *safety* × *drug*, *F*(4,1964) = 11.43, *p* < 0.001, η_p_^2^ = 0.02, and *group* × *safety* interactions, *F*(4,1968) = 4.13, *p* = 0.002, η_p_^2^ = 0.008. Bonferroni-corrected post hoc tests (five comparisons) revealed that the MI+ group was significantly more likely than the MI− group to agree that MDMA should be tested for its medicinal value, *F*(1,491) = 7.78, *p* = 0.005, η_p_^2^ = 0.016, that the government should fund studies exploring medicinal use of psilocybin and MDMA, *F*(1,491) = 14.72, *p* < 0.001, η_p_^2^ = 0.029, and that psilocybin should be granted medical treatment status for use in licensed facilities under the supervision and care of medical practitioners, *F*(1,491) = 8.99, *p* = 0.003, η_p_^2^ = 0.018 (see Figure 3(E) and (F)). Overall, participants were significantly more likely to indicate that MDMA can be addictive compared to psilocybin, *F*(1,491) = 250.79, *p* < 0.001, η_p_^2^ = 0.338. Relative to MDMA, participants across both groups were significantly more likely to agree that psilocybin can be safely enjoyed when used recreationally, akin to alcohol or tobacco, *F*(1,491) = 89.43, *p* < 0.001, η_p_^2^ = 0.154, should be tested for its medicinal value, *F*(1,491) = 93.93, *p* < 0.001, η_p_^2^ = 0.161, should receive government funding for studies, *F*(1,491) = 65.36, *p* < 0.001, η_p_^2^ = 0.117, and should be granted medical treatment status, *F*(1,491) = 97.73, *p* < 0.001, η_p_^2^ = 0.166.

## Discussion

This study compared attitudes toward psychedelics and psychedelic-assisted therapy among Australians with and without self-reported mental illness. Our findings reveal a nuanced landscape of perceptions, whereby individuals with mental illness showed a more favorable stance toward the legalization and therapeutic use of psychedelics than those without. Participants with mental illness were more open to the use of psychedelics, advocating for their legalization and emphasizing potential benefits over risks. This aligns with findings by Corrigan et al. (2022), who reported a similar openness among mental health consumers in Ireland. Participants’ support for government-funded research into psychedelics further underscores a societal shift toward exploring alternative mental health treatments, mirroring the attitudes of Australian psychiatrists advocating for more research into the safety and efficacy of psychedelics under medical supervision (Berger and Fitzgerald, 2023; Grover et al., 2023).

Both groups exhibited similar levels of knowledge about psychedelics. Classic psychedelics, such as LSD and psilocybin, were frequently recognized. Conversely, our study also highlights a significant knowledge gap regarding psychedelics, with participants from both groups misidentifying substances like opium and ketamine as psychedelics. While ketamine is not a classic psychedelic, its hallucinogenic effects may contribute to this confusion, especially among the general public. This highlights the need for enhanced education on how psychoactive substances are perceived and understood. Our finding echoes Žuljević et al. (2022), pointing to a widespread misunderstanding about what constitutes a psychedelic substance. The poor recognition of peyote, mescaline and DMT may relate to a lack of cultural exposure in Australia, as these substances are traditionally used by Indigenous cultures in the Americas (George et al., 2020). The inverse relationship between self-rated and actual knowledge about psychedelics among individuals with mental illness underscores the importance of public education in correcting misconceptions about these substances.

Despite greater familiarity with MDMA compared with psilocybin, both groups perceived greater therapeutic potential for psilocybin. Relative to MDMA, participants were more willing to take prescribed psilocybin, cease psychiatric medications in preparation for psilocybin-assisted therapy and participate in psilocybin-assisted psychotherapy. Psilocybin was also seen as safer, while MDMA was viewed as more addictive. This suggests a complex interplay between drug reputation, perceived safety and therapeutic potential. This preference may be influenced by the cultural and historical context of MDMA as a ‘party drug’, as well as by media portrayals and anti-drug campaigns in Australia, which have historically targeted MDMA (Australian Government Department of Health, 2020; Wyndham, 2021). Conversely, the perception of psilocybin as safer and more connected to nature may reflect its less stigmatized status and its association with traditional and indigenous uses (George et al., 2020).

Self-rated knowledge of psychedelics, factual understanding of these substances and personal experience with them predicted participants’ perspectives on legalization, perceived benefits, risks and willingness to engage with psychedelics. This underscores the significant role that knowledge and personal experience play in shaping opinions about these substances. It suggests that educational interventions could profoundly influence public perception and acceptance of psychedelic use. Žuljević et al. (2022) identified similar relationships between knowledge of psychedelics and attitudes, further emphasizing the potential impact of targeted information dissemination on shaping societal attitudes toward psychedelics.

Our study’s reliance on self-reported diagnoses and the cross-sectional design limits the generalizability of our findings and introduces potential biases. The overrepresentation of middle-aged, educated participants does not reflect the general Australian population or mental health consumers (Australian Bureau of Statistics, 2023; Australian Institute of Health and Welfare, 2024), further narrowing the scope of our findings. This highlights the need for more inclusive research that captures the diversity of the Australian population and mental health consumers. In addition, the proportion of participants who had used psychedelics was greater than that previously reported in the general Australian population (Australian Institute of Health and Welfare, 2024). The group of individuals with mental illness had more experience with substance use than the group without mental illness, which may have biased their views toward the legalization of psychedelics. Indeed, Australians with personal experience of using illegal substances are more likely to support their legalization (Weatherburn et al., 2022), and previous psychedelic use is generally associated with positive attitudes toward psychedelics (Corrigan et al., 2022; Grover et al., 2023). Our study did not collect data about Indigenous status, ethnicity or sexual orientation—groups known to utilize mental health services at a higher proportional rate (Australian Bureau of Statistics, 2023; Australian Institute of Health and Welfare, 2024). Future research should aim to address these limitations by incorporating a broader demographic spectrum, including Aboriginal and Torres Strait Islander peoples, individuals from various ethnic backgrounds and members of the LGBTQIA+ community.

Our findings contribute to the growing body of literature supporting a more nuanced understanding of public attitudes toward psychedelics and their potential role in mental health treatment. While there is a clear interest and support among potential mental health service users for the therapeutic use of psychedelics, significant work remains to educate the public and healthcare professionals about these substances. As Australia and other countries continue to explore the possibilities of psychedelic-assisted therapy, it is imperative that future research and policymaking are informed by a comprehensive and inclusive understanding of public attitudes and knowledge about psychedelics.

## Supplemental Material

sj-docx-1-anp-10.1177_00048674241261779 – Supplemental material for Attitudes toward psychedelics and psychedelic-assisted therapy among potential mental health service users and the general population in Australia Supplemental material, sj-docx-1-anp-10.1177_00048674241261779 for Attitudes toward psychedelics and psychedelic-assisted therapy among potential mental health service users and the general population in Australia by Zohaib Nadeem, Stephen Parker, Hugh McGovern and Lena KL Oestreich in Australian & New Zealand Journal of Psychiatry

sj-docx-2-anp-10.1177_00048674241261779 – Supplemental material for Attitudes toward psychedelics and psychedelic-assisted therapy among potential mental health service users and the general population in Australia Supplemental material, sj-docx-2-anp-10.1177_00048674241261779 for Attitudes toward psychedelics and psychedelic-assisted therapy among potential mental health service users and the general population in Australia by Zohaib Nadeem, Stephen Parker, Hugh McGovern and Lena KL Oestreich in Australian & New Zealand Journal of Psychiatry

sj-docx-3-anp-10.1177_00048674241261779 – Supplemental material for Attitudes toward psychedelics and psychedelic-assisted therapy among potential mental health service users and the general population in Australia Supplemental material, sj-docx-3-anp-10.1177_00048674241261779 for Attitudes toward psychedelics and psychedelic-assisted therapy among potential mental health service users and the general population in Australia by Zohaib Nadeem, Stephen Parker, Hugh McGovern and Lena KL Oestreich in Australian & New Zealand Journal of Psychiatry

sj-png-4-anp-10.1177_00048674241261779 – Supplemental material for Attitudes toward psychedelics and psychedelic-assisted therapy among potential mental health service users and the general population in Australia Supplemental material, sj-png-4-anp-10.1177_00048674241261779 for Attitudes toward psychedelics and psychedelic-assisted therapy among potential mental health service users and the general population in Australia by Zohaib Nadeem, Stephen Parker, Hugh McGovern and Lena KL Oestreich in Australian & New Zealand Journal of Psychiatry

sj-png-5-anp-10.1177_00048674241261779 – Supplemental material for Attitudes toward psychedelics and psychedelic-assisted therapy among potential mental health service users and the general population in Australia Supplemental material, sj-png-5-anp-10.1177_00048674241261779 for Attitudes toward psychedelics and psychedelic-assisted therapy among potential mental health service users and the general population in Australia by Zohaib Nadeem, Stephen Parker, Hugh McGovern and Lena KL Oestreich in Australian & New Zealand Journal of Psychiatry

sj-png-6-anp-10.1177_00048674241261779 – Supplemental material for Attitudes toward psychedelics and psychedelic-assisted therapy among potential mental health service users and the general population in Australia Supplemental material, sj-png-6-anp-10.1177_00048674241261779 for Attitudes toward psychedelics and psychedelic-assisted therapy among potential mental health service users and the general population in Australia by Zohaib Nadeem, Stephen Parker, Hugh McGovern and Lena KL Oestreich in Australian & New Zealand Journal of Psychiatry
