# Supplementary material for: Attitudes toward psychedelics and psychedelic-assisted therapy among potential mental health service users and the general population in Australia
Source: Aust N Z J Psychiatry. 2024 Jun 22;58(10):904–13. doi: 10.1177/00048674241261779 (PMC11420588; doi:10.1177/00048674241261779)
Supplement: sj-docx-2-anp-10.1177_00048674241261779 – Supplemental material for Attitudes toward psychedelics and psychedelic-assisted therapy among potential mental health service users and the general population in Australia [file sj-docx-2-anp-10.1177_00048674241261779.docx]

| **Table 2.** Knowledge of psychedelics and other substances | | | | | |
| --- | --- | --- | --- | --- | --- |
| **Substance** | **Response, n (%)** | | | | **Comparison** |
|  | *Mental illness* | | *No mental illness* | |  |
| **Psychedelics** | **Correctly identified as a psychedelic** | **Incorrectly identified as non-psychedelic** | **Correctly identified as a psychedelic** | **Incorrectly identified as non-psychedelic** |  |
| Lysergic acid diethylamide (LSD) | 288 (88.9) | 36 (11.1) | 138 (78.9) | 37 (21.1) | *χ^2^*(1) = 9.16, *p* = 0.088* |
| MDMA (ecstasy) | 223 (68.8) | 101 (31.2) | 133 (76) | 42 (24) | *χ^2^*(1) = 0.36, *p* = 1 |
| Psilocybin | 267 (82.4) | 56 (17.3) | 125 (71.4) | 50 (28.6) | *χ^2^*(1) = 3.19, *p* = 1 |
| DMT | 176 (54.3) | 147 (45.4) | 78 (44.6) | 97 (55.4) | *χ^2^*(1) = 4.47, *p* = 0.77* |
| Mescaline | 131 (40.4) | 192 (59.3) | 54 (30.9) | 121 (69.1) | *χ^2^*(1) = 4.57, *p* = 0.7* |
| Peyote | 171 (52.8) | 152 (46.9) | 71 (40.6) | 104 (59.4) | *χ^2^*(1) = 6.95, *p* = 0.18* |
| Ibogaine | 31 (9.6) | 292 (90.1) | 11 (6.3) | 164 (93.7) | *χ^2^*(1) = 1.61, *p* = 1 |
| **Non-psychedelics** | **Incorrectly identified as a psychedelic** | **Correctly identified as a non-psychedelic** | **Incorrectly identified as a psychedelic** | **Correctly identified as a non-psychedelic** |  |
| Opium | 116 (35.8) | 208 (64.2) | 74 (42.3) | 101 (57.7) | *χ^2^*(1) = 2.03, *p* = 1 |
| Methamphetamine | 76 (23.5) | 247 (76.2) | 58 (33.1) | 117 (66.9) | *χ^2^*(1) = 5.33, *p* = 0.46* |
| Heroin | 55 (17) | 269 (83) | 46 (26.3) | 129 (73.7) | *χ^2^*(1) = 6.1, *p* = 0.31* |
| Cocaine | 53 (16.4) | 271 (83.6) | 42 (24) | 133 (76) | *χ^2^*(1) = 4.31, *p* = 0.87 |
| Dextroamphetamine | 52 (16) | 272 (84) | 37 (21.1) | 138 (78.9) | *χ^2^*(1) = 2.01, *p* = 1 |
| Ketamine | 139 (42.9) | 184 (56.8) | 57 (32.6) | 118 (67.4) | *χ^2^*(1) = 5.21, *p* = 0.51* |
| Gamma-hydroxybutyrate (GHB) | 89 (27.5) | 234 (72.2) | 49 (28) | 126 (72) | *χ^2^*(1) = 0.01, *p* = 1 |
| Rohypnol | 48 (14.8) | 275 (84.9) | 22 (12.6) | 153 (87.4) | *χ^2^*(1) = 0.49, *p* = 1 |
| Oxycodone | 51 (15.7) | 272 (84) | 25 (14.3) | 150 (85.7) | *χ^2^*(1) = 0.2, *p* = 1 |
| Haloperidol | 25 (7.7) | 298 (92) | 7 (4) | 168 (96) | *χ^2^*(1) = 2.64, *p* = 1 |
| Mexazolam | 18 (5.6) | 305 (94.1) | 13 (7.4) | 162 (92.6) | *χ^2^*(1) = 0.67, *p* = 1 |
| Phenobarbital | 45 (13.9) | 278 (85.8) | 14 (8) | 161 (92) | *χ^2^*(1) = 0.62, *p* = 1 |
| Modafinil | 15 (4.6) | 308 (95.1) | 9 (5.1) | 166 (94.9) | *χ^2^*(1) = 3.19, *p* = 1 |
| Imipramine | 13 (4) | 310 (95.7) | 6 (3.4) | 169 (96.6) | *χ^2^*(1) = 0.11, *p* = 1 |
| Digoxin | 21 (6.5) | 302 (93.2) | 5 (2.9) | 170 (97.1) | *χ^2^*(1) = 3.05r, *p* = 1 |

*Note.* P-values are Bonferroni corrected for 22 comparisons. *uncorrected p-value is significant.
