# Supplementary figures and images for: Attitudes toward psychedelics and psychedelic-assisted therapy among potential mental health service users and the general population in Australia
Source: Aust N Z J Psychiatry. 2024 Jun 22;58(10):904–13. doi: 10.1177/00048674241261779 (PMC11420588; doi:10.1177/00048674241261779)

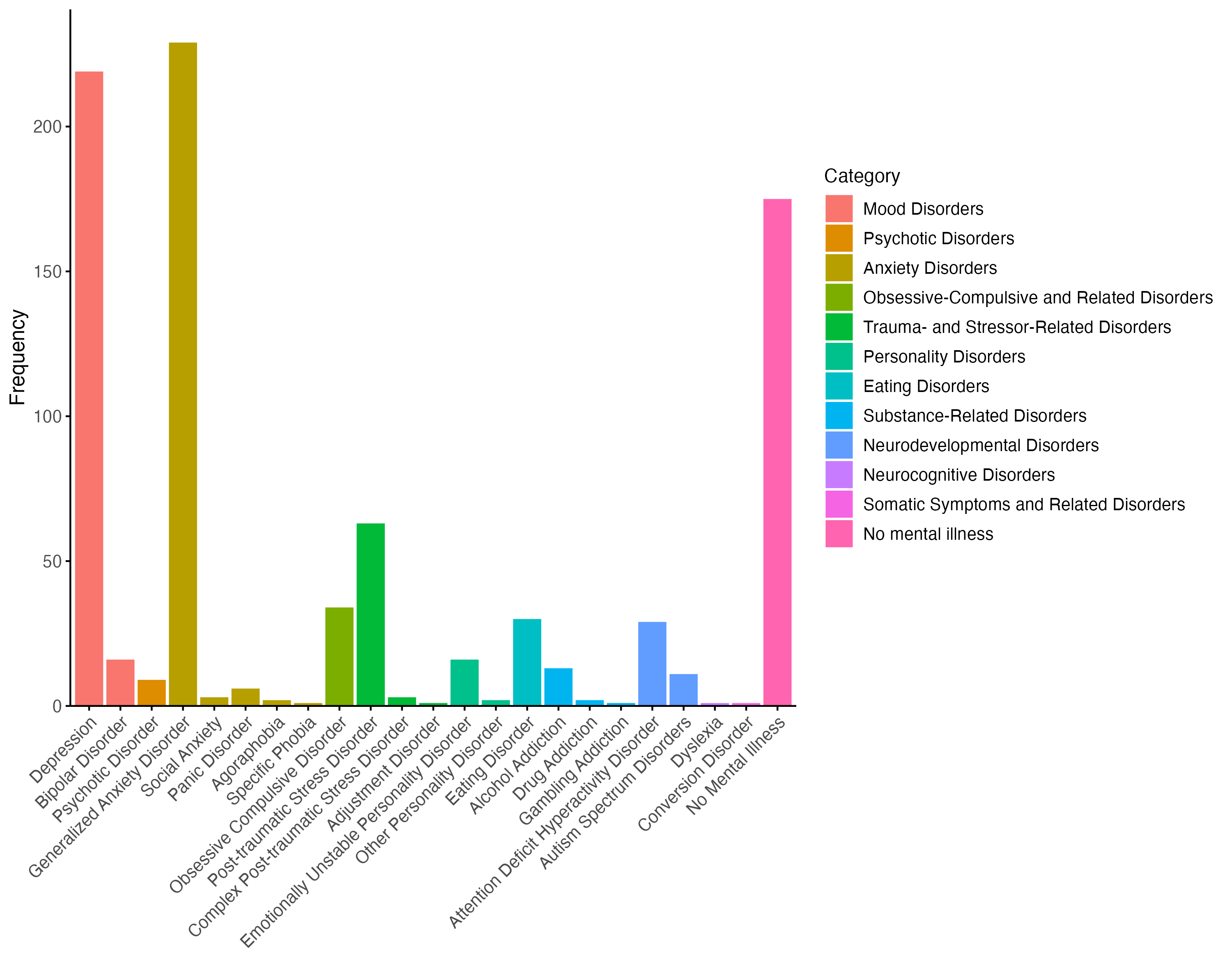

Supplement: sj-png-4-anp-10.1177_00048674241261779 – Supplemental material for Attitudes toward psychedelics and psychedelic-assisted therapy among potential mental health service users and the general population in Australia [file sj-png-4-anp-10.1177_00048674241261779.png]

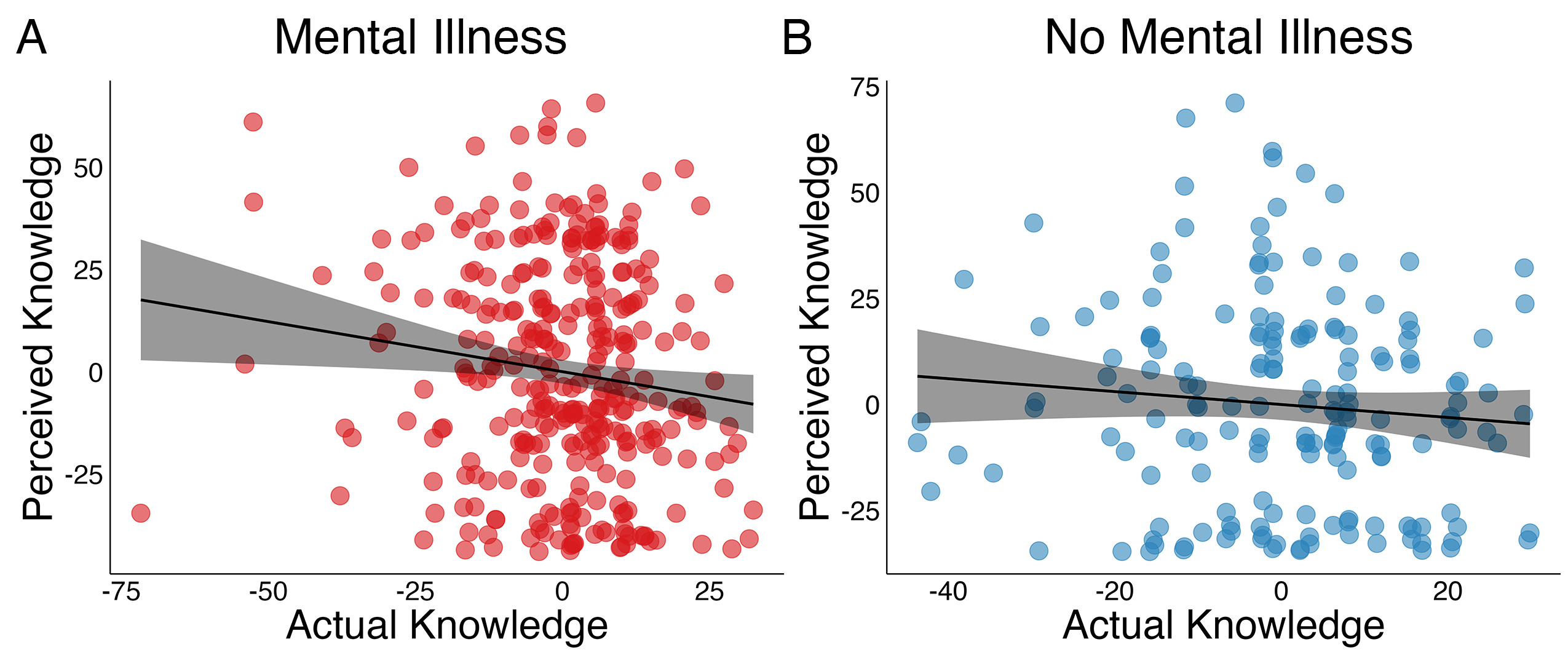

Supplement: sj-png-5-anp-10.1177_00048674241261779 – Supplemental material for Attitudes toward psychedelics and psychedelic-assisted therapy among potential mental health service users and the general population in Australia [file sj-png-5-anp-10.1177_00048674241261779.png]

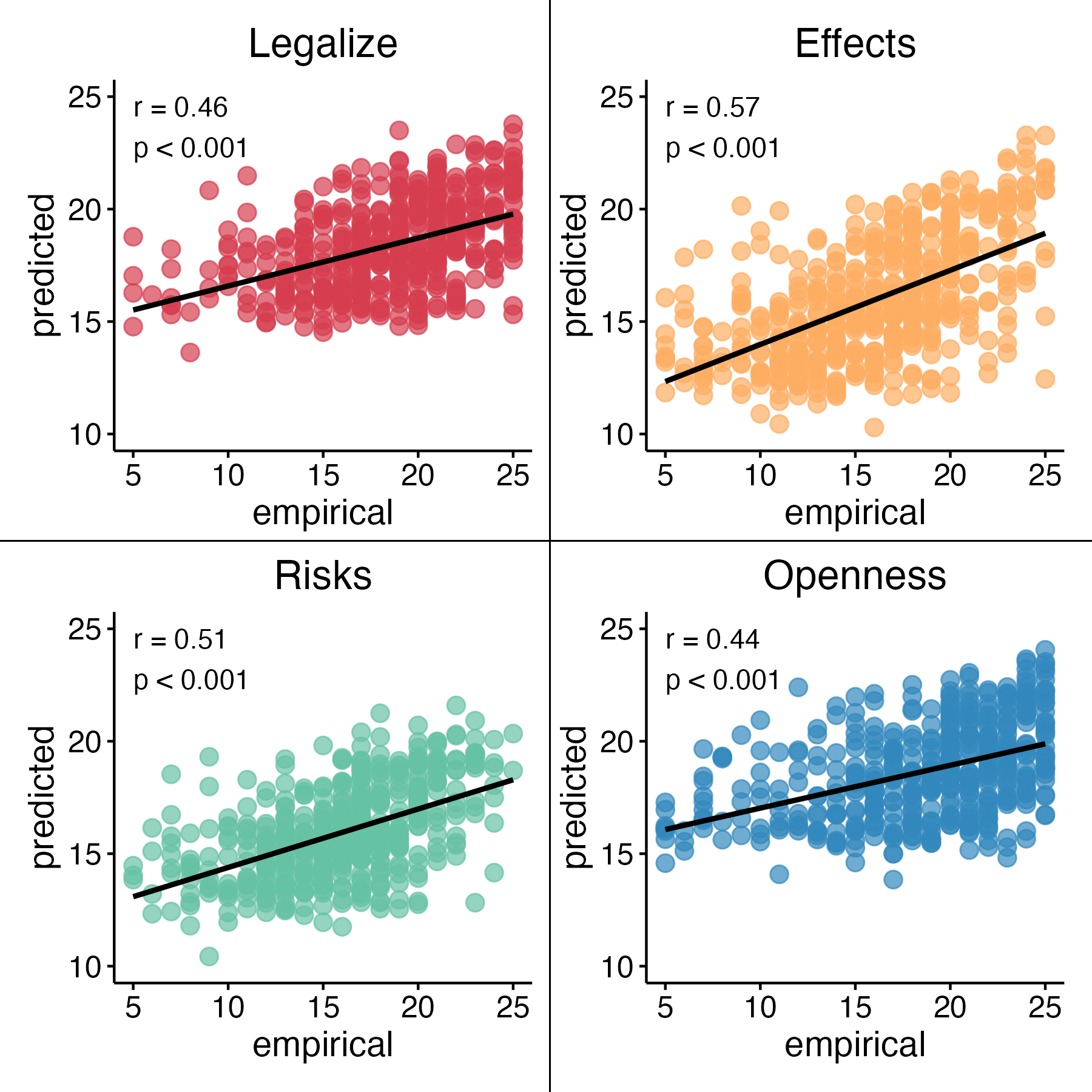

Supplement: sj-png-6-anp-10.1177_00048674241261779 – Supplemental material for Attitudes toward psychedelics and psychedelic-assisted therapy among potential mental health service users and the general population in Australia [file sj-png-6-anp-10.1177_00048674241261779.png]
